# Reprogramming mechanisms influence the maturation of hematopoietic progenitors from human pluripotent stem cells

**DOI:** 10.1038/s41419-018-1124-6

**Published:** 2018-10-24

**Authors:** Hye-Ryeon Heo, Haengseok Song, Hye-Ryun Kim, Jeong Eun Lee, Young Gie Chung, Woo Jin Kim, Se-Ran Yang, Kye-Seong Kim, Taehoon Chun, Dong Ryul Lee, Seok-Ho Hong

**Affiliations:** 1 Department of Internal Medicine, School of Medicine, Kangwon National University, Chuncheon, Republic of Korea; 2 Department of Biomedical Science, College of Life Science, CHA University, Seongnam, Republic of Korea; 3 Research Institute for Stem Cells, CHA Health Systems, Los Angeles, CA 90036 USA; 4 Department of Thoracic and Cardiovascular Surgery, School of Medicine, School of Medicine, Kangwon National University, Chuncheon, Republic of Korea; 5 Department of Biomedical Science, College of Medicine, Hanyang University, Seoul, 04763 Republic of Korea; 6 Department of Biotechnology, College of Life Science and Biotechnology, Korea University, Seoul, Republic of Korea

## Abstract

Somatic cell nuclear transfer (SCNT) or the forced expression of transcription factors can be used to generate autologous pluripotent stem cells (PSCs). Although transcriptomic and epigenomic comparisons of isogenic human NT-embryonic stem cells (NT-ESCs) and induced PSCs (iPSCs) in the undifferentiated state have been reported, their functional similarities and differentiation potentials have not been fully elucidated. Our study showed that NT-ESCs and iPSCs derived from the same donors generally displayed similar in vitro commitment capacity toward three germ layer lineages as well as proliferative activity and clonogenic capacity. However, the maturation capacity of NT-ESC-derived hematopoietic progenitors was significantly greater than the corresponding capacity of isogenic iPSC-derived progenitors. Additionally, donor-dependent variations in hematopoietic specification and commitment capacity were observed. Transcriptome and methylome analyses in undifferentiated NT-ESCs and iPSCs revealed a set of genes that may influence variations in hematopoietic commitment and maturation between PSC lines derived using different reprogramming methods. Here, we suggest that genetically identical iPSCs and NT-ESCs could be functionally unequal due to differential transcription and methylation levels acquired during reprogramming. Our proof-of-concept study indicates that reprogramming mechanisms and genetic background could contribute to diverse functionalities between PSCs.

## Introduction

Human pluripotent ESCs, which are successfully derived by isolating an inner cell mass from a viable blastocyst, are allogeneic^1^. To overcome the issue of allogeneity, two innovative reprogramming approaches for converting somatic cells into PSCs were evaluated. The first approach involved the cellular reprogramming of somatic cells to pluripotency by the forced expression of four transcription factors (TFs), which resulted in the generation of iPSCs^2,3^. More recently, we and two other research groups independently reported the establishment of diploid pluripotent ESCs by transferring the nucleus of fetal and adult fibroblasts into enucleated oocytes^4–6^. These two reprogramming methods yield autologous PSCs, which could be suitable for the development of patient-specific cell therapies that do not cause immune rejection^7^. Thus, determining whether iPSCs and NT-ESCs are genetically safe and functionally competent is critical prior to their use in personalized regenerative medicine.

Recent achievements in the generation of human NT-ESCs have enabled the performance of detailed genetic and epigenetic comparisons between genetically matched human iPSCs and NT-ESCs, eliminating the genetic heterogeneity among the PSC lines compared^8,9^. These studies revealed that both cell types contained a similar number of coding mutations and variations in de novo copy number that were not detected in the donor somatic cells. Interestingly, Ma et al. reported the incomplete epigenetic reprogramming of iPSCs, and suggested that the transcriptional and epigenetic signatures of NT-ESCs are more similar to ESCs compared to iPSCs. Contrary to this finding, Johannesson et al. reported that the number of epigenetic changes between the two cell types was equivalent. The controversy between the two studies might be due to the use of different reprogramming methods or to the involvement of somatic cell donors with different potentials. Hence, understanding the fundamental states of NT-ESCs and iPSCs and determining the functional features of isogenic iPSCs and NT-ESCs are critical issues that must be addressed prior to their therapeutic application^10^.

In this study, we generated isogenic sets of human NT-ESCsand iPSCs derived from different donors and compared their fundamental properties, including proliferation, clonogenicity, and heterogeneity in the undifferentiated state. Further, we first evaluated the in vitro potential of the isogenic pairs to differentiate into three germ layer lineages.

## Materials and methods

### Human SCNT-ESC and iPSC lines

CHA-hES NT2, 4, 5, and 8 (hereafter named NT, NT2, NT4, NT5, and NT8) for human SCNT-ESCs and iPS-NT2-S4, iPS-NT4-S1, iPS-NT4-E15, iPS-NT5-S9, and iPS-NT8-S1 (hereafter named iPS2, iPS4, iPS4-Epi, iPS5, and iPS8) for isogenic iPSCs were used in this study. Human ESC line (CHA-hES 15, ESC) was used as a control. All these cell lines were initially produced in CHA Stem Cell Institute, CHA University, Seoul, South Korea. For human SCNT-ESC derivation, the procedures were described in the previous report^4^. iPSC2, 4, 5, and 8 were generated using Sendai virus-based vectors, which express OCT4, SOX2, KLF4, and c-MYC (Cyto-Tune^TM^-iPS Reprogramming kit; Invitrogen) according to the manufacturer’s protocol. Transgene and virus-free iPSC4-Epi was generated using episomal reprogramming vector, which express OCT4, SOX2, KLF4, LIN28, and L-MYC (Epi5^TM^ Episomal iPSC Reprogramming Kit; Invitrogen). Somatic donor for NT4 and iPS4 was a healthy male donor (35 years old). Somatic donor for NT5 and iPS5 was a female patient with age-related macular degeneration (73 years old).

### Characterization of human NT-ESCs and iPSCs

Immunocytochemistry (ICC) and reverse transcription-polymerase chain reaction (RT-PCR) were performed to confirm hESC-specific marker expression. For ICC, antibodies against OCT3/4 (Santa Cruz, 1:100), SSEA-4 (Cell Signaling, 1:100), TRA 1-60 (Millipore, 1:100), TRA 1-81 (Millipore, 1:100), and Alexa Flour® 555 goat anti-mouse IgG antibody (Molecular probes, 1:200) were used, and cell nuclei were co-stained with DAPI (Vector Laboratories). For RT-PCR, we confirmed the expression of *OCT4*, *NANOG*, and *SOX2* genes using following primer sequences: *OCT4* (F) 5′-GCAATTTGCCAAGCTCCTGAAGCAG-3′, (R) 5′-CATAGCCTGGGGTACCAAAAT GGGG-3′ (536 bp); *NANOG* (F) 5′-TGAACCTCAGCTACAAACAG-3′, (R) 5′-TGG TGGTAGGAAGAGTAAAG-3′ (153 bp); *SOX2* (F) 5′-AGCTACAGCATGATGCAGGA-3′, (R) 5′-GGTCATGGAGTTGTACTGCA-3′ (125 bp); and *GAPDH* (F) 5′-TGAAGG TCGGAGTCAACGGATTTGGT-3′, (R) 5′-CATGTGGGCCATGAGGTCCACCAC-3′ (983 bp)/(F) 5′-AGAAGGCTGGGGCTCATTTG-3′, (R) 5′-AGGGGCCATCCACAG TCTTC-3′ (258 bp) as an internal control.

The differentiation capacity of NT-ESC and iPSC lines was confirmed by embryoid body (EB) formation in vitro and teratoma formation in vivo. For EB formation, NT-ESCs and iPSCs were cultured in suspension without human bFGF for 2 weeks, and then the differentiation of EBs into three germ layers was confirmed by immunohistochemistry (IHC) and RT-PCR. For IHC, antibodies against AFP (Alpha-1-Fetoprotein; Dako, 1:100) for endoderm, SMA (alpha smooth muscle actin; Abcam, 1:100) for mesoderm, and TUJ1 (B-tubulin; Covance, 1:100) for ectoderm were used. Alexa Flour^®^ 555 goat anti-rabbit IgG antibody (Molecular probes) was used as a secondary antibody (1:200) and nuclei were stained with DAPI (Vector Laboratories). Primer sequences for RT-PCR are listed in Supplementary Table 1. For teratoma formation, approximately 1×10^5^ of undifferentiated NT-ESCs and iPSCs were injected into a testicle of NOD/SCID male mouse. For each NT-ESC and iPSC line, at least three animals were used. After 12–16 weeks, teratomas were excised, fixed in paraformaldehyde, embedded in paraffin, sectioned, and then analyzed histologically after staining.

### Chromosome analysis

Chromosome analyses for both NT-ESCs and iPSCs were performed using a standard protocol. Metaphase spreads were stained with a GTG (G-bands by trypsin using Giemsa)-banding technique. Twenty metaphases were analyzed and karyotyped by two cytogenetics experts. The ideogram was produced by the Ikaros karyotyping system (MetaSystems, Germany).

### Genomic DNA fingerprinting

DNA fingerprinting of donor fibroblasts, donor oocytes, and NT-ESCs was performed by using AmpFISTR^®^ identifier kit (Applied Biosystems). PCR reactions were performed according to the manufacturer’s protocol. The PCR products were loaded on an ABI 3130 genetic analyzer (Applied Biosystems) and analyzed by the GeneMapper^®^ID Software v3.2.1 (Applied Biosystems).

### Maintenance of human NT-ESCs and iPSCs

All human PSC lines used in this study were maintained in mTeSR1 serum-free medium (Stem Cell Technologies) on Matrigel (BD Biosciences)-coated tissue culture plates. They were subcultured once every 5 days by mechanical dissociation.

### BrdU incorporation and cell cycle analysis

Undifferentiated human ESCs, iPSCs, and NT-ESCs were incubated with 10 μM of bromodeoxyuridine (BrdU) for 4 h at 37 °C and then fixed with 70% ethanol containing 50 mM glycine for 20 min. Cell cycle analysis was performed using allophycoerythrin (APC)-BrdU Flow Kit (BD Pharmingen) following the manufacturer’s instruction. Cells were stained with APC-conjugated anti-BrdU antibody and 7-amino actinomycin (7ADD). Flow cytometric analysis was performed by using a FACSCanto II flow cytometer (BD Biosciences), and FlowJo software (Tree Star) was used to derive the fraction of cells distributed throughout various phases of the cell cycle.

### Clonogenic assay

Dissociation of human iPSCs and NT-ESCs were performed as previously shown^2^. For quantitative comparison of clonogenic potential between iPSCs and NT-ESCs, we seeded 10,000 cells into a six-well tissue culture plate coated with Matrigel in mTeSR1 medium supplemented with 10 μM Rho-associated protein kinase (ROCK) inhibitor for the initial 24 h. On the next day, all non-adherent cells were removed and fresh medium without ROCK inhibitor was added. The numbers of colonies were counted after 7 days of culturing.

### Alveolar epithelial progenitor cell differentiation

The stepwise alveolar epithelial progenitor cell (AEC) differentiation was performed as previously described with minor modification^3^. Briefly, undifferentiated iPSC and NT-ESC colonies were prepared with low density of less than 10 colonies per well of a six-well tissue culture plate coated with Matrigel. When the colonies grew up to approximately 1 mm in diameter, the mTeSR1 medium was replaced by RPMI1640 medium containing 1× B27 supplement, activin A (100 ng/mL; R&D systems), 1 μM of CHIR99021 (Axon Medchem), and 0.125 mM sodium butyrate (Sigma) and cultured for 6 days to induce to definitive endoderm (DE) cells. The DE cells were differentiated to ventralized anterior foregut endodermal (VAFE) cells as a AECs via AFE cells by sequential exposure to two different combinations of external factors for 8 days (100 ng/mL of noggin and 10 μM SB-431542 for 4 days, followed by 100 ng/mL of BMP4, 1 μM all-*trans* retinoic acid, and 1.5 μM CHIR99021 for 4 days). On day 14, VAFE cells were further differentiated to alveolar and distal airway epithelial (ADAE) cells by culturing in Ham’s F12 medium supplemented with KGF10 (100 ng/mL), 50 nM dexamethasone, 0.1 mM 8-br-cAMP, and 0.1 mM 3-isobutyl-1-methylxantine. Induction efficiency was assessed by observing the localization and frequencies of AEC and ADAE cell markers (NKX2.1, EPCAM, CPM, and SFTP-B) on days 14 and 25 using immunostaining and flow cytometry.

### Neural differentiation

Human iPSCs and NT-ESCs were induced to neural progenitor cells (NPCs) using serum-free STEMdiff^TM^ Neural Induction Medium (Stem Cell Technologies) according to the manufacturer’s instruction. Briefly, undifferentiated iPSC and NT-ESC colonies were prepared with low density of less than 10 colonies per well of a six-well tissue culture plate coated with Matrigel. When the colonies grew up to approximately 1 mm in diameter, we replaced mTeSR1 medium with Neural Induction Medium and cultured for 9 days. NPC markers (SOX1, SOX2, NESTIN, and GFAP) were used to assess the induction efficiency on day 9 by flow cytometry.

### Hematopoietic differentiation

A stepwise direct hematopoietic differentiation was performed as previously described with slight modifications^4^. Briefly, undifferentiated iPSC and NT-ESC colonies were prepared with low density (less than 10 colonies in a single well of six-well tissue culture plates). When the colonies grew up to approximately 1 mm in diameter, the hemogenic specification began with them exposed to Stemline II serum-free medium supplemented with Insulin-Transferrin-Selenium and BMP4 (20 ng/mL) for 4 days, followed by treatment with SCF (50 ng/mL) and VEGF (40 ng/mL) for 2 days. On day 6, the cultures were given fresh hematopoietic induction medium supplemented with hematopoietic cocktail (50 ng/mL SCF, 10 ng/mL TPO, 50 ng/mL IL3, 50 ng/mL FLT-3L, and 50 ng/mL G-CSF) as described in Fig. 4a and cultured for 11 days. All cytokines were purchased from R&D Systems. The commitment capacity was assessed by measuring the frequencies of CD34- and CD45-positive cells on day 17 by flow cytometry.

### Colony-forming units assay

Colony-forming units (CFU) assay was performed by plating 10,000 hematopoietic progenitor cells into methylcellulose H4434 (Stem Cell Technologies) supplemented with SCF (50 ng/mL), IL3 (10 ng/mL), EPO (3 U/mL), and GM-CSF (10 ng/mL). After incubation for 14 days at 37 °C in 5% CO_2_, hematopoietic cell clusters were counted using standard morphological criteria.

### Flow cytometric analysis

Single-cell suspensions were harvested from undifferentiated or differentiated iPSCs and NT-ESCs. Undifferentiated cultures were dissociated with cell dissociation medium (CDM; Invitrogen) for 20 min at 37 °C. Differentiated cells were treated with collagenase IV for 2 h, followed by treatment with CDM for 20 min in a 37 °C incubator. The cells were filtered through a 70 μm cell strainer and incubated for 30 min at 4 °C with following anti-human antibodies: OCT4 (BD Biosciences), SSEA-3 (BD Biosciences), SSEA-4 (BD Biosciences), CD34 (Miltenyi), CD45 (BD Biosciences), E-Cadherin (BD Biosciences), SOX1 (BD Biosciences), SOX2 (BD Biosciences), NESTIN (BD Biosciences), GFAP (BD Biosciences), NKX2.1 (Abcam), CPM (Abcam), and EPCAM (Santa Cruz). For intracellular staining of OCT4, SOX1, SOX2, and NKX2.1, the cells were fixed and permeabilized using the Cytofix/Cytoperm buffer (BD Biosciences). Dead cells were excluded by 7AAD staining. Flow cytometric analysis was performed by using a FACSCanto II flow cytometer and acquired data were analyzed by the FlowJo software.

### Immunofluorescence staining

Undifferentiated PSCs and AECs were rinsed with phosphate-buffered saline (PBS) and fixed with 4% paraformaldehyde (Sigma) for 20 min at room temperature. After washing with PBS, cells were permeabilized with 0.5% saponin (Sigma) in PBS containing 1% bovine serum albumin for 10 min and then blocked with 10% normal rabbit or donkey serum for 30 min. The following primary and secondary antibodies were used: goat anti-OCT3/4 (Santa Cruz), mouse anti-CPM (Abcam), rabbit anti-NKX2.1 (Abcam), mouse anti-EPCAM (Santa Cruz), rabbit anti-goat Alexa Flour 594 (Invitrogen), goat anti-mouse Alexa Fluor 594 (Invitrogen), donkey anti-mouse Alexa Fluor 594 (Invitrogen), and donkey anti-rabbit Alexa Fluor 488 (Invitrogen). Nuclei were stained with prolong gold DAPI with antifade (Invitrogen). Immunofluorescence signals were observed and photographed with an Olympus IX51 microscope equipped with a photometrix Cool Snap HQ2 camera using Image-Pro 3DA vs. 6.0. software.

### qRT-PCR

Total RNA was extracted from undifferentiated PSCs and their derivatives using RNeasy kit (Qiagen). First strand cDNA was synthesized from 500 ng of total RNA using TOPscript^TM^ RT DryMIX kit (Enzynomix). The cDNA was then subjected to qPCR using AccuPower^®^ PCR PreMix reagents (Bioneer Corp). Primer sequence is listed in Supplementary Table 1. Melting curve analyses were performed to confirm the presence of a single amplicon with correct size and absence of nonspecific bands. The expression levels of each gene were normalized to GAPDH and the relative quantification was conducted using the comparative CT method according to the manufacturer’s instructions (Applied Biosystems).

### Microarray analysis

Total RNA was extracted with TriZol from undifferentiated hESC, hiPSC, and hNT-ESC lines. The quality of total RNA was evaluated using an Agilent 2100 Bioanalyzer. Agilent Human Gene expression 4x44K V2 microarray (Agilent Technologies, Santa Clara, CA, USA) was hybridized with appropriate probe at the facility of E-Biogen (Seoul, Korea). The hybridization images were analyzed by an Agilent’s DNA microarray Scanner and normalization steps were performed using GeneSpring GX 7.3 software. Hierarchical clustering with the average linkage method was performed with MeV4.9.0 software. Genes were classified into specific biological processes by gene ontology and expression profiles of gene clusters were visualized as heat maps with the MultiExperiment Viewer program. The heat map signals were generated by the log2 transformed human iPSC/human NT-ESC intensity ratio to compare expression level of genes.

### Methylation analysis

Genomic DNA extracted from human iPSC and NT-ESC lines was assessed for DNA methylation using Human CpG island 244k microarray. The array was entrusted by E-Biogen (Seoul, Korea). The raw intensities of the scanned image for both human iPSC-Cy5 and NT-ESC-Cy3 channels were extracted and normalized according to the manufacturer’s instructions (Agilent Technology, USA). The heat maps and boxplots show the signal intensity ratios generated by the log2 transformed human iPSC/human NT-ESC intensity to compare methylation landscapes of CpG sites on the promoter region of genes classified into specific biological processes between isogenic cell lines.

### Statistical analysis

Values for all measurements were presented as mean ± SD. Statistical significance was determined using Student’s *t*-test and *p* < 0.05 was considered statistically significant.

## Results

### Intrinsic proliferative and clonogenic potentials of isogenic PSC pairs are not altered by the reprogramming process

**Fig. 1:**
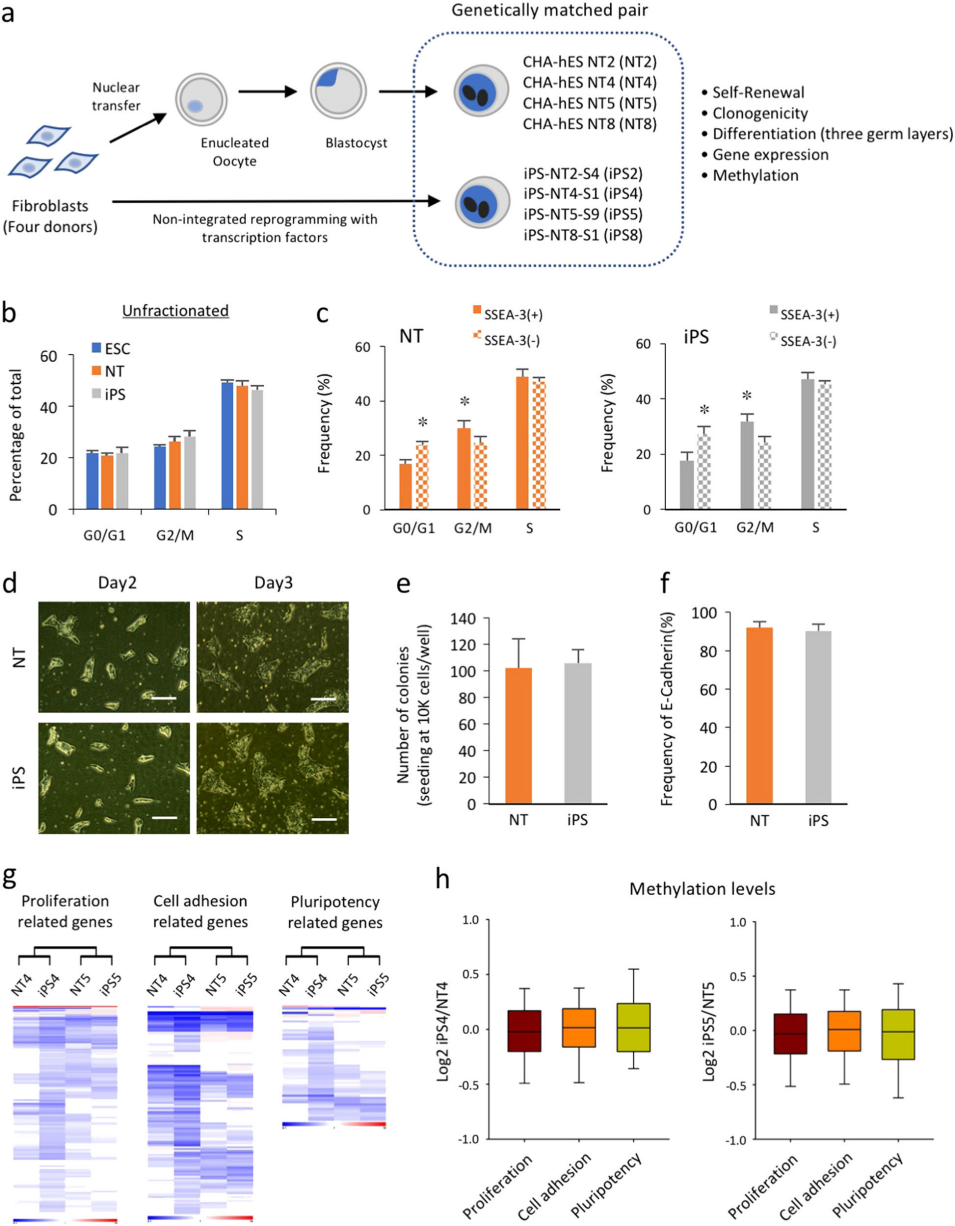
Isogenic iPSCs and NT-ESCs exhibit similar proliferative and clonogenic potentials. **a** A schematic diagram of the study design. Genetically matched iPSCs and NT-ESCs were derived from the dermal fibroblasts of four different donors. **b** Graphic representation of the cell cycle compartments in unfractionated iPSC, NT-ESC, and ESC cultures using BrdU incorporation assay. **c** Flow cytometry analysis of BrdU incorporation gated on the SSEA-3(+) and SSEA-3(−) subpopulations within the iPSC and NT-ESC cultures. *p < 0.05. **d** Representative images of the colonies that formed from dissociated single cells and the regenerated colonies counted 7 days post seed are shown. Scale bar, 100 µm. **e** Quantitative comparison of the clonogenic capacity of the iPSCs and NT-ESCs. **f** The percentages of E-cadherin in the undifferentiated iPSC and NT-ESC cultures determined by flow cytometry. **g** Heat maps representing the mRNA expression levels of genes associated with proliferation, cell adhesion, and pluripotency in the isogenic iPSCs and NT-ESCs. **h** Boxplots representing the degree of promoter gene methylation within each category. All bars indicate the mean±SD from three independent experiments

To determine if genetically matched human iPSCs and NT-ESCs are functionally equivalent, we generated four pairs of isogenic iPSCs (iPS2, 4, 5, and 8) and NT-ESCs (NT2, 4, 5, and 8) using dermal fibroblasts from four different donors (Fig. 1a). All of the cell lines exhibited typical morphology, expressed pluripotency markers (Supplementary Fig. 1a), and retained normal karyotypes for up to 30 passages (Supplementary Fig. 1b). Initial experiments were mainly performed with two isogenic pairs (4 and 5) and the other two lines (2 and 8) were subsequently generated and used for following investigation^4,11^. The presence of a short tandem repeat at the human autosomal loci and at the 1 X/Y locus confirmed that the genotypes of each isogenic pair were genetically identical to each other and to the donor cells (Supplementary Fig. 2a). We also verified that the mitochondrial genomes of NT cell lines are matched those of the donor, confirming that the cytoplasm of these NT-ESC lines originated from the donor oocytes (Supplementary Fig. 2b). The pluripotency of all cell lines was demonstrated by their ability to spontaneously differentiate into all three embryonic germ layers both in vitro (Supplementary Figs. 3 and 4a) and in an in vivo teratoma formation assay (Supplementary Fig. 4b). In addition, all of the cell lines were maintained under the same serum- and feeder-free conditions to eliminate potential variability due to different culture conditions. The cell lines were morphologically indistinguishable and strongly expressed pluripotency markers including OCT4, SSEA-3, and SSEA-4 (Supplementary Figs. 5a, b). The unsupervised hierarchical clustering of the isogenic iPSC and NT-ESC pairs clearly indicated that the mRNA landscapes of the undifferentiated pluripotent cells were primarily donor-dependent (Supplementary Fig. 5c).

Similar to other human PSCs, both of the undifferentiated isogenic pairs exhibited a very short G1 phase that accounted for approximately 15–20% of the cell cycle^12^. Likewise, both cell types exhibited similar BrdU incorporation rates (iPSCs, 46.4±1.5% vs NT-ESCs, 48.2±1.8%) and comparable cell cycle compartment distributions (Fig. 1b). Previous reports demonstrated functional differences between subpopulations within the PSC cultures by separating the different cell types based on the expression of surface markers including SSEA-3 and c-KIT^13,14^. Accordingly, we fractionated the isogenic iPSCs and NT-ESCs based on SSEA-3 expression and detected a significantly higher proportion of SSEA-3(+) cells in the G2/M phase compared with SSEA-3(−) cells in both of the isogenic iPSC and NT-ESC cultures (Fig. 1c), which indicated the presence of heterogeneous subsets with differing cell cycle activity within the cultures. We also found that dissociated iPSCs and NT-ESCs both propagated as undifferentiated cultures that were morphologically identical to the original cultures and that exhibited a similar clonogenic capacity (Fig. 1d, e). Previous studies have reported that the expression of E-Cadherin plays a role in improving the survival and clonogenicity of dissociated human PSCs^15^. As expected, both isogenic pairs strongly expressed E-cadherin, which supported their clonogenic capacity (Fig. 1f). These results clearly indicated that functional similarities in pluripotency, proliferation, and cell adhesion exist between isogenic PSCs. To evaluate whether the observed functional equivalence of the isogenic iPSCs and NT-ESCs was reflected in the transcription and/or methylation levels in the undifferentiated cultures, we analyzed their transcription and methylation profiles. The percentage of differentially expressed genes and differentially methylated regions (DMRs) were similar between the isogenic pairs (Supplementary Figs. 6a, b), and the DMR distribution among the different chromosomes was comparable (Supplementary Figs. 6c, d). Furthermore, the overall methylation levels of the gene promoter regions were likewise similar, suggesting relatively equivalent epigenetic landscapes. In accord with the epigenetic similarities, comparable heat map patterns representing the groups of genes involved in proliferation, cell adhesion, and pluripotency reinforced the biological similarities between the isogenic PSCs (Fig. 1g, h). In these biological processes, reprogramming procedure did not produce inverse correlation between mRNA expression and promoter methylation of genes (detailed values summarized in Supplementary Excel File 1). Collectively, these results indicated that the reprogramming process does not significantly alter the intrinsic proliferative and clonogenic potentials of PSCs with identical genetic backgrounds.

### Isogenic iPSCs and NT-ESCs exhibit similar alveolar epithelial cell and neuronal differentiation capacities

**Fig. 2:**
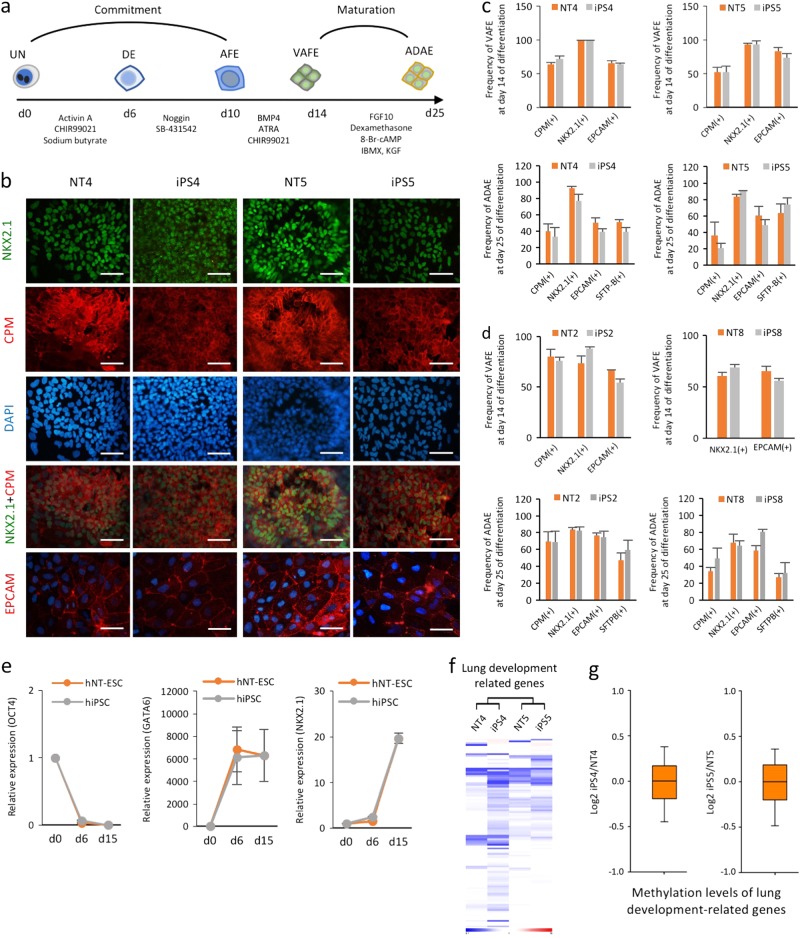
Isogenic iPSCs and NT-ESCs show similar alveolar epithelial cell (AEC) differentiation potentials. **a** Schematic diagram of serum- and feeder-free multistep AEC differentiation from human iPSCs and NT-ESCs. DE definitive endoderm, AFE anterior foregut endoderm. **b** Immunofluorescence staining for CPM, NKX2.1, and EPCAM in AECs on day 14 of differentiation. Scale bar, 200 μm. **c, d** Flow cytometry analysis of cells harvested on days 14 and 25 showing the frequencies of specific markers for VAFE and ADAE cells, respectively. The induction efficiency of VAFE and ADAE cells was determined by measuring the percentages of CPM(+), NKX2.1(+), EPCAM(+), and SFTP-B(+) cells by flow cytometry on days 14 and 25, respectively. **e** AEC differentiation was measured by gradually reducing *OCT4* and increasing the lineage specific markers (*GATA6* and *NKX2.1*). The relative expression levels of target genes are normalized to GAPDH in each well. Values are relative to day 0 (day 0 = 1). **f** Heat maps representing the RNA expression patterns of genes associated with lung development in isogenic iPSCs and NT-ESCs. **g** Boxplots representing the mean lung development-related gene promoter methylation levels in each isogenic pair. All bars indicate the mean±SD from three independent experiments

Next, we explored whether isogenic human iPSCs and NT-ESCs possess comparable potentials for in vitro differentiation into the three specific germ layer lineages. First, we evaluated the in vitro potential of the isogenic pairs to differentiate into an AEC lineage to determine endodermal potential. Using a previously reported stepwise induction protocol^16^, we compared the induction efficiency in VAFE and ADAE cells on days 14 and 25, respectively, using several specific markers (NKX2.1, CPM, EPCAM, and SFTP-B) (Fig. 2a). Following immunofluorescence staining, we found that AEC markers (NKX2.1, CPM, and EPCAM) were strongly expressed on day 14 (Fig. 2b). The frequencies of both VAFE and ADAE cells did not differ significantly between the isogenic iPSCs and NT-ESCs on days 14 and 25 (Fig. 2c, d). AEC differentiation resulted in the time-dependent downregulation of *OCT4* and the upregulation of AEC-related genes including *GATA6* and *NKX2.1* (Fig. 2e), indicating that the AEC lineage commitment of iPSCs and NT-ESCs followed similar trajectories in response to external stimuli. We then explored the fundamental nature of lung developmental-related genes in the isogenic pairs in the undifferentiated state at both the transcriptional and epigenetic levels. The mRNA expression profiles (Fig. 2f) and promoter methylation levels (Fig. 2g) of genes involved in lung development in the undifferentiated isogenic PSCs did not indicate a bias for the AEC lineage (Supplementary Excel file 1). These results indicated that isogenic iPSCs and NT-ESCs in undifferentiated cultures possess similar AEC commitment and maturation capacities in the absence of a genetic or epigenetic predisposition to the AEC lineage.

**Fig. 3:**
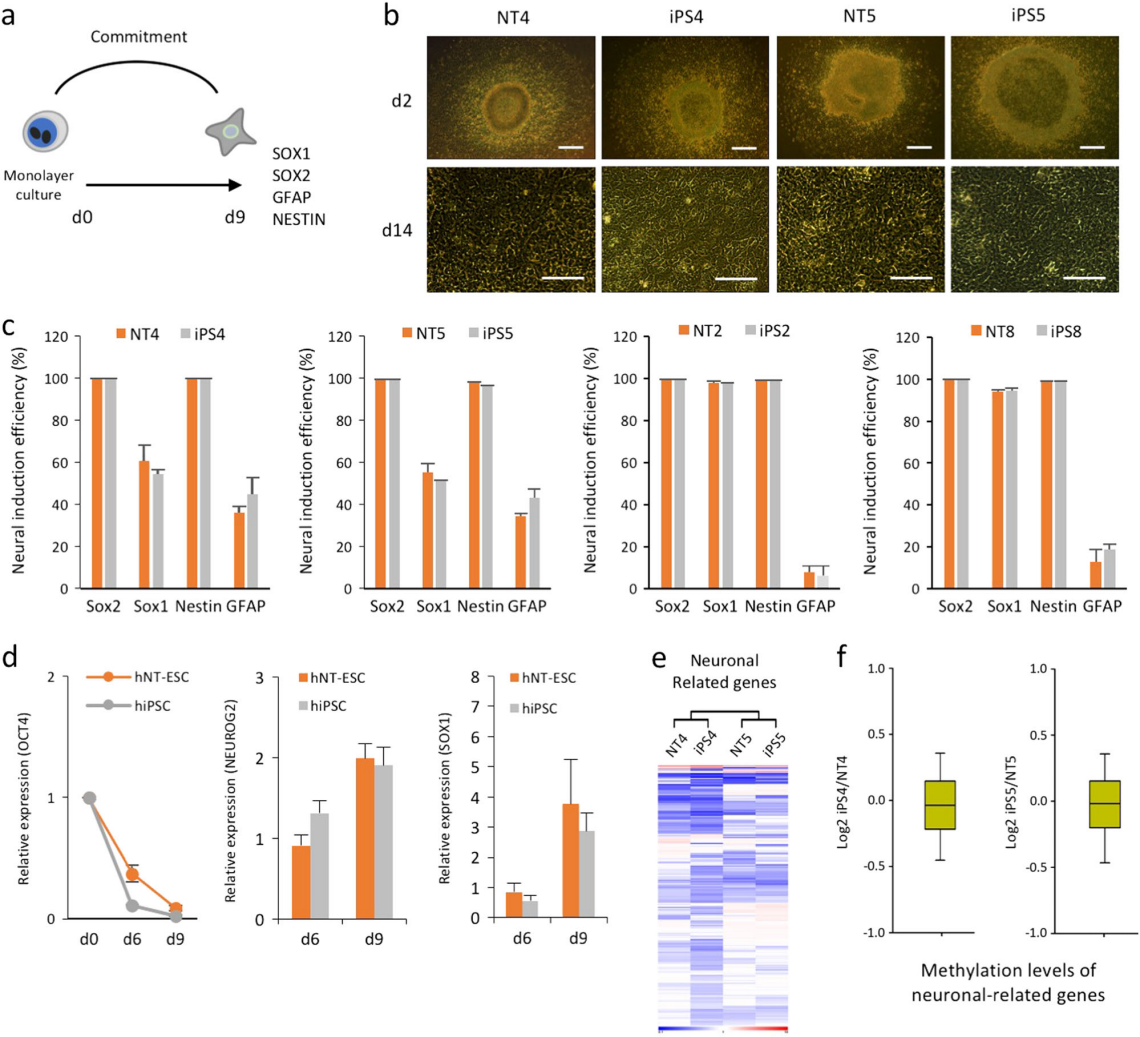
Isogenic iPSCs and NT-ESCs exhibit similar neuronal differentiation capacities. **a** A schematic of serum- and feeder-free NPC differentiation from human iPSCs and NT-ESCs. **b** Representative images of neural differentiation. Scale bar, 100 μm. **c** Flow cytometry analysis of cells harvested on day 9 showing the frequencies of specific NPC markers. The induction efficiency of neuronal cells determined by measuring the percentages of SOX2(+), SOX1(+), NESTIN(+), and GFAP(+) cells by flow cytometry on day 9. **d** NPC differentiation measured by gradually reducing *OCT4* and increasing the neuronal lineage markers (*NEUROG2* and *SOX1*). The relative expression levels of *OCT4* are normalized to GAPDH in each well. Values are relative to day 0 (day 0 = 1). **e** Heat maps representing the RNA expression patterns of genes associated with neuronal differentiation in both isogenic iPSCs and NT-ESCs. **f** Boxplots representing the mean neuronal-related gene promoter methylation levels in each isogenic pair. All bars indicate the mean±SD from three independent experiments

We likewise compared the differentiation potential of isogenic iPSCs and NT-ESCs to NPCs to determine ectodermal lineage potential using an established serum-free neural induction protocol (Fig. 3a). All of the undifferentiated cell line colonies exhibited a similar morphology to NPCs during differentiation (Fig. 3b). Isogenic iPSCs and NT-ESCs likewise displayed similar NPC marker frequencies (SOX2, SOX1, NESTIN, and GFAP) on day 9 of differentiation. In addition, no variation in neural differentiation potential was observed in the PSCs derived from two donors (Fig. 3c). NPC differentiation resulted in the concurrent upregulation of neural-related genes (*NEUROG2* and *SOX1*) and the time-dependent downregulation of *OCT4* (Fig. 3d), indicating that the induction of neural lineage commitment in iPSCs and NT-ESCs using specific soluble stimuli was similar. Furthermore, the mRNA expression profiles (Fig. 3e) and promoter methylation levels (Fig. 3f) of genes involved in neuronal differentiation in the undifferentiated isogenic iPSCs and NT-ESCs indicated the absence of a genetic or epigenetic predisposition to the neural lineage (Supplementary Excel File 1).

### Donor-dependent variations in hematopoietic commitment capacity between human PSC lines

**Fig. 4:**
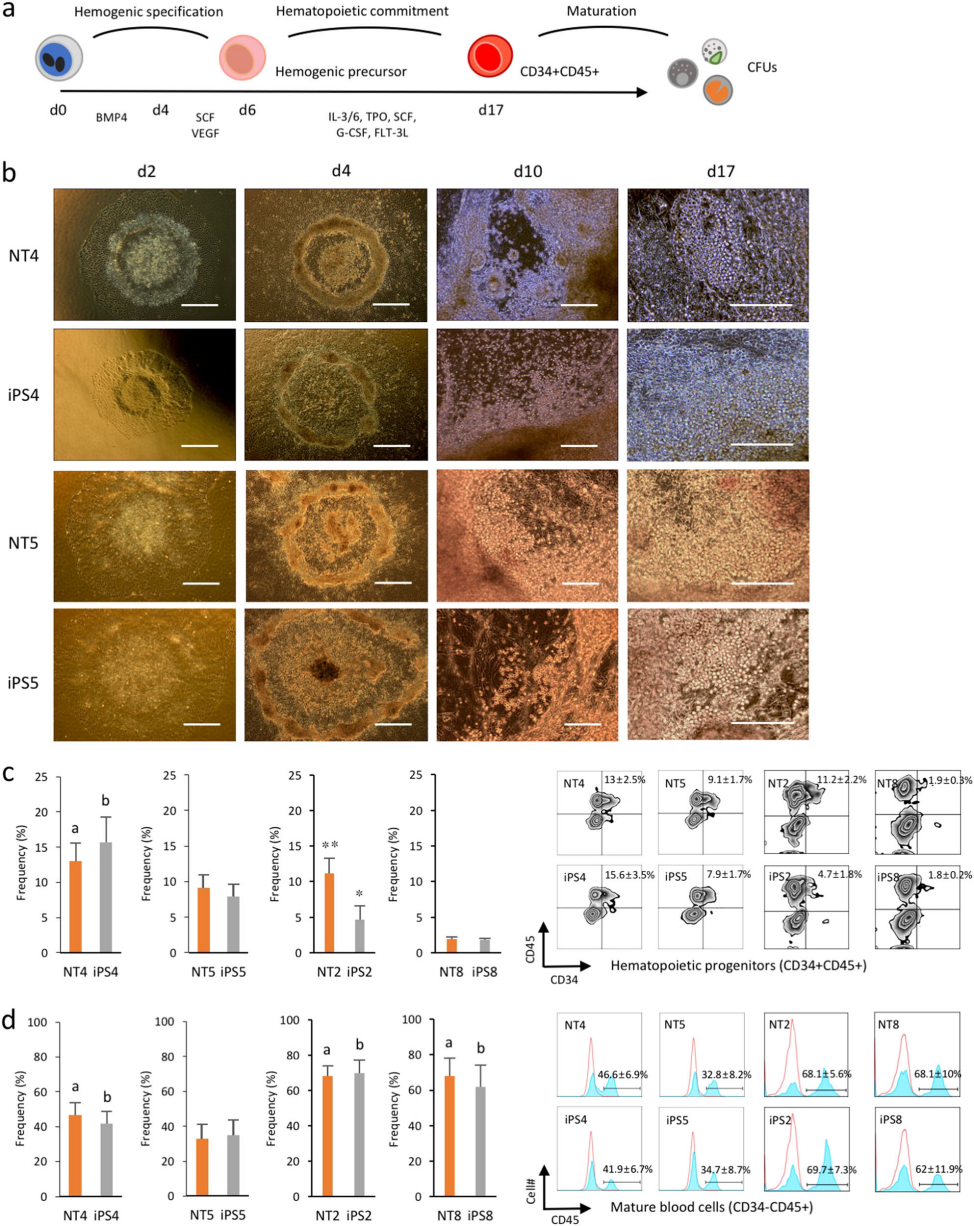
Donor-dependent variations in hematopoietic commitment capacity between human PSC lines. **a** Schematic diagram of serum- and feeder-free stepwise hematopoietic induction of human iPSCs and NT-ESCs. Flow cytometry analysis of committed hematopoietic progenitors (CD34+CD45+) and mature blood (CD34−CD45+) cells on day 17. **b** Representative images at different stages of hematopoietic differentiation. Scale bar, 100 μm. **c, d** Flow cytometry analysis of cells harvested on day 17 showing the frequencies of hematopoietic progenitor and mature blood cells. ^a^*p* < 0.05 (vs NT5), ^b^*p* < 0.05 (vs iPS5), *p < 0.05 (vs iPS8), **p < 0.01 (vs NT8). Both floating and attached cells were harvested to measure the efficiency if hematopoietic differentiation. All bars indicate the mean±SD from three independent experiments

Lastly, we attempted to induce the differentiation of the isogenic pairs to the hematopoietic lineage to determine mesodermal potential. We employed a directed, stepwise differentiation protocol as previously described with some modifications^17^ (Fig. 4a). Following hematopoietic induction using BMP4, colonies from all of the cell lines exhibited a similar plateau-like central area surrounded by monolayer cells. Floating blood cells emerged from the hematopoietic cell clusters along the plateau area margins during the hematopoietic commitment phase (Fig. 4b). We assessed the commitment capacity of all of the cell lines by measuring the primitive hematopoietic progenitor (CD34+CD45+ or CD34+CD43+) and mature hematopoietic (CD34−CD45+) cell frequencies on day 17. CD43 was previously identified as a hematopoietic progenitor marker for human ESC-derived hematopoiesis in vitro^18^. No significant differences in the CD34+CD45+, CD34+CD43+, or CD34−CD45+ subset frequencies were detected between the isogenic iPSCs and NT-ESCs (Fig. 4c, d and Supplementary Figs. 7a, b). However, NT4 and iPS4 yielded significantly more hematopoietic progenitors and mature blood cells than NT5 and iPS5. NT2 and iPS2 generated higher levels of hematopoietic progenitors than NT8 and iPS8. In addition, both NT2/iPS2 and NT8/iPS8 pairs exhibited significantly higher frequencies of CD45+ mature blood cells compared with NT5 and iPS5. This finding was consistent with those from recent studies demonstrating donor-dependent variations in hematopoietic differentiation potential among hPSC lines^19,20^. To further understand the underlying mechanisms that influence donor-dependent variations in hematopoietic differentiation at the molecular level, we compared the expression profiles of hematopoietic-related genes in undifferentiated NT4 and NT5 and iPS4 and iPS5. We found that several genes were up or downregulated in NT4 and iPS4 compared to NT5 and iPS5, and the observed differences in expression levels were validated using qPCR (Supplementary Figs. 7c, d). In PSCs, RUNX1 expression, a master regulator of hematopoietic stem cell emergence, enhances the conversion of undifferentiated PSCs to hematopoietic progenitor cells^21^. GYPA (CD235) is a specific marker for human erythroid precursors^22^. These findings suggest that the increased expression of hematopoietic-related genes such as RUNX1 and GYPA in NT4 and iPS4 may prime undifferentiated cells toward hematopoietic or erythrocyte lineage commitment or maturation.

### Differential transcription and methylation levels acquired during reprogramming may influence the hematopoietic maturation capacity of isogenic NT-ESCs and iPSCs

**Fig. 5:**
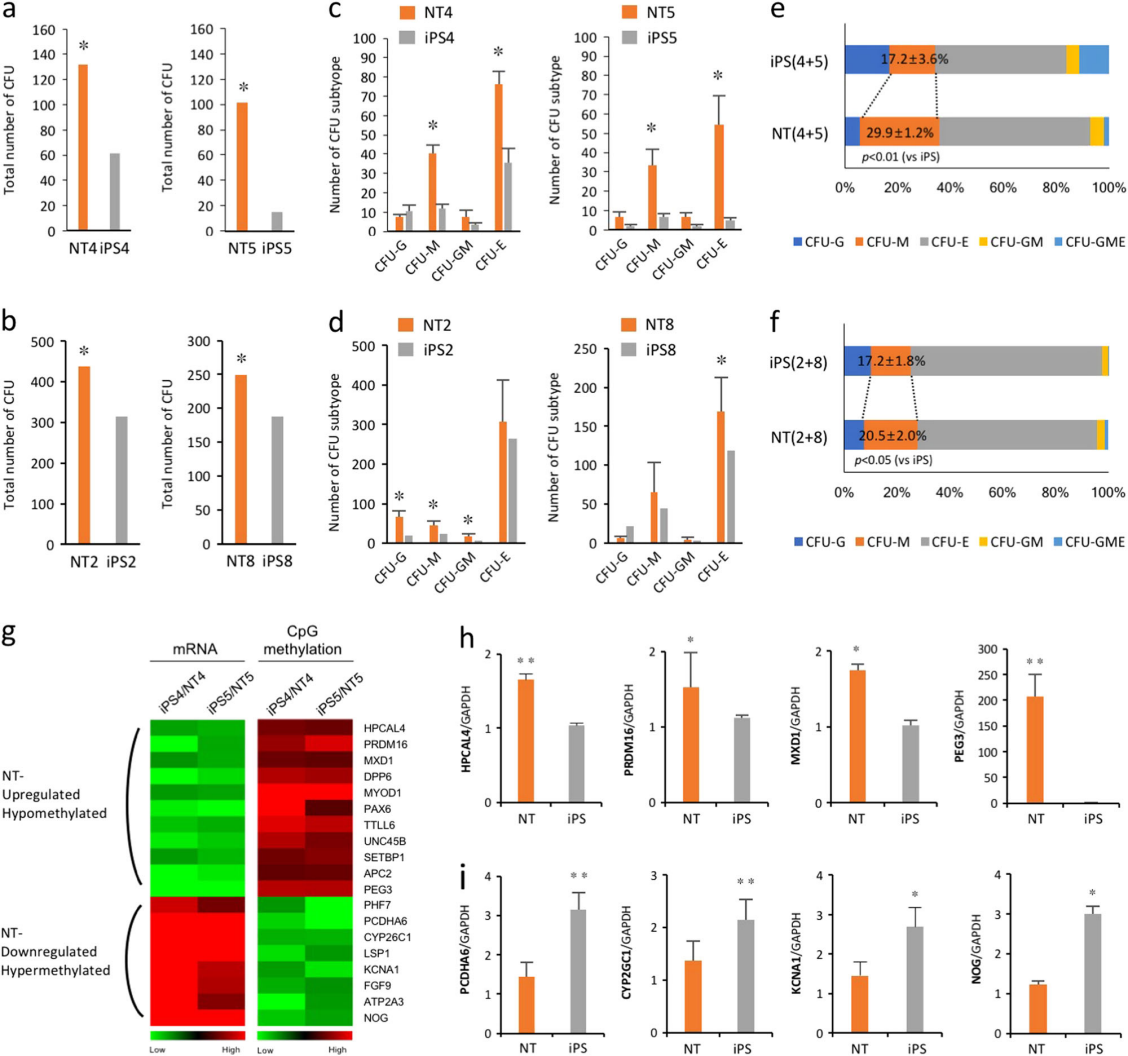
Differential transcription and methylation levels in genes related to hematopoietic development during reprogramming may influence the maturation capacity of isogenic NT-ESCs and iPSCs. **a–f** Assessment of hematopoietic progenitor capacity by counting the number (**a–d**) and distribution of CFU subtypes (**e, f**). CFU-E, erythroid; CFU-M, macrophage; and CFU-G, granulocytes; CFU-GME, granulocytes/macrophage/erythroid. *p < 0.05. **g** Heat maps representing up- and downregulated genes in NT lines compared with iPSC lines and the promoter methylation status of corresponding genes. **h**, **i** Verification of the transcriptomic and methylomic analysis of selected genes using qPCR. *p < 0.05, **p < 0.01. All bars indicate the mean±SD from three independent experiments

To evaluate the apparent functional inequality between the isogenic pairs with respect to the maturation capacity of the hematopoietic progenitors, an equal number of hematopoietic progenitors were plated on methylcellulose and the colony-forming potential was measured (Supplementary Fig. 7e). Interestingly, the hematopoietic progenitors derived from the NT-ESC lines produced more colony-forming units (CFUs) (Fig. 5a, b) and gave rise to a greater number of CFU-macrophage (CFU-M) and CFU-erythrocytes (CFU-E) than the iPSC lines (Fig. 5c, d). In addition, CFU assay showed a significant increase of the percentage of CFU-M colonies in the NT-ESC lines compared to the iPS lines (Fig. 5e, f)

The functional advantage of human NT-ESCs with regard to the maturation capacity of the hematopoietic progenitors in this study was reinforced by similar findings reported in mouse isogenic NT-ESCs and iPSCs^23^. To investigate the molecular signatures associated with the greater maturation capacity of NT-ESCs, we analyzed the transcription profiles of hematopoietic-related genes in the isogenic pairs (NT4 vs iPS4 and NT5 vs iPS5), and detected higher (11 genes) and lower (8 genes) expression among the genes in NT-ESCs. Furthermore, a strong inverse correlation was detected between the gene expression profiles and the methylation status of their promoter regions (Fig. 5g–i). Numerous reprogramming methods have been developed to generate iPSCs and may lead to different transcript levels of the same genes which can have specific biological functions^24^. Thus, we generated iPSCs (iPS4-Epi) with non-integrating episomal vectors from the donor cells for iPS4 generation and then compared the expression levels of the selected genes between iPS4-Epi and NT4. Interestingly, iPS4-Epi showed the similar expression patterns as seen in iPS4 (Supplementary Figs. 8a, b), indicating that this phenomenon may not reprogramming method-dependent. Taken together, our results indicate that hematopoietic commitment capacity is not positively correlated with maturation capacity, which might be due to the differential transcription and methylation of genes related to hematopoietic development during reprogramming.

## Discussion

Recent reports have suggested that the cell fate potential and functional variations among human ESCs and iPSCs primarily depend on the donors rather than on the origin of the cell type^19,20,25^. In this study, we also determined that genetically identical NT-ESCs and iPSCs are generally functionally equivalent. However, our data clearly demonstrated that stage-specific variations during differentiation, such as the maturation capacity of hematopoietic progenitors, might be due to different reprogramming systems. The predisposition potentials could be influenced by different genetic and/or epigenetic landscapes acquired during reprogramming. Therefore, monitoring transcriptome and epigenome alterations during reprogramming by NT or forced TF expression is required to understand the resulting differentiation capacity^26^.

In our study, we have identified a set of potential genes that might influence variations in hematopoietic maturation between isogenic NT-ESCs and iPSCs. Some of the genes upregulated in NT-ESCs are known to enhance self-renewal and the differentiation of murine and human hematopoietic stem/progenitor cells. For example, PRDM16 induced by HOXA9 enhances the self-renewal of hematopoietic and myeloid progenitor cells^27^. HOXA9 also promotes hematopoietic differentiation in human ESCs^28^, indicating that the HOXA9–PRDM16 axis may play a crucial role in hematopoietic differentiation. MDX1 serves as a transcriptional regulator during the megakaryocytic and granulocytic differentiation of hematopoietic and myeloid progenitors^29–31^. Interestingly, the expression of NOG, a BMP4 antagonist, was notably downregulated in NT-ESCs, suggesting that increased BMP4 action may promote the self-renewal and maturation of hematopoietic progenitors in NT-ESCs. Thus, further genetic functional studies will be required to define the precise role of these genes in determining the differentiation propensity of human PSC lines toward hematopoietic lineage.

Although comparable biological features of undifferentiated isogenic NT-ESCs and iPSCs have been reported at the genetic and epigenetic levels, data evaluating their differentiation potentials are limited. A previous report indicated that the ability of mouse NT-ESCs to form hematopoietic colonies is greater than the corresponding capacity of isogenic iPSCs^32^. Recently, Zhao et al.^33^ demonstrated the functional similarity of cardiomyocytes and endothelial cells derived from isogenic human iPSCs and NT-ESCs. However, both prior studies only evaluated the cell fate potential of the mesodermal lineage. Therefore, the precise evaluation of the functional equivalence of the isogenic pairs requires a comparison of a broad spectrum of fundamental features including the differentiation of three germ layer lineages and self-renewal capacity. To our knowledge, we are the first to evaluate the functional equivalence of isogenic human NT-ESCs and iPSCs regarding the lineage specificity of all three germ layers, and to provide evidence that reprogramming mechanisms could contribute to a functional bias toward differentiation lineage specificity between PSCs. The isogenic iPSC and NT-ESC pairs used in this study were derived from one cell type (dermal fibroblast). Thus, it is critical to note that future studies involving the establishment and testing of large cohorts of isogenic pairs derived from various cell types are required to strengthen our findings. More importantly, in vivo studies involving humanized animal and disease models will be required to determine the regenerative potential and immunogenicity of isogenic NT-ESCs and iPSCs.

## Electronic supplementary material

Supplementary Information

Dataset 1
