# Supplementary material for: Reprogramming mechanisms influence the maturation of hematopoietic progenitors from human pluripotent stem cells
Source: Cell Death Dis. 2018 Oct 24;9(11):1090. doi: 10.1038/s41419-018-1124-6 (PMC6200746; doi:10.1038/s41419-018-1124-6)
Supplement: Supplementary file 1 — Supplementary Information [file 41419_2018_1124_MOESM1_ESM.docx]

**Supplementary Information**

**Reprogramming mechanisms influence the maturation of hematopoietic progenitors from human pluripotent stem cells**

Hye-Ryeon Heo, Haengseok Song, Hye-Ryun Kim, Jeong Eun Lee, Young Gie Chung, Woo Jin Kim, Se-Ran Yang, Kye-Seong Kim, Taehoon Chun, Dong Ryul Lee, Seok-Ho Hong

**
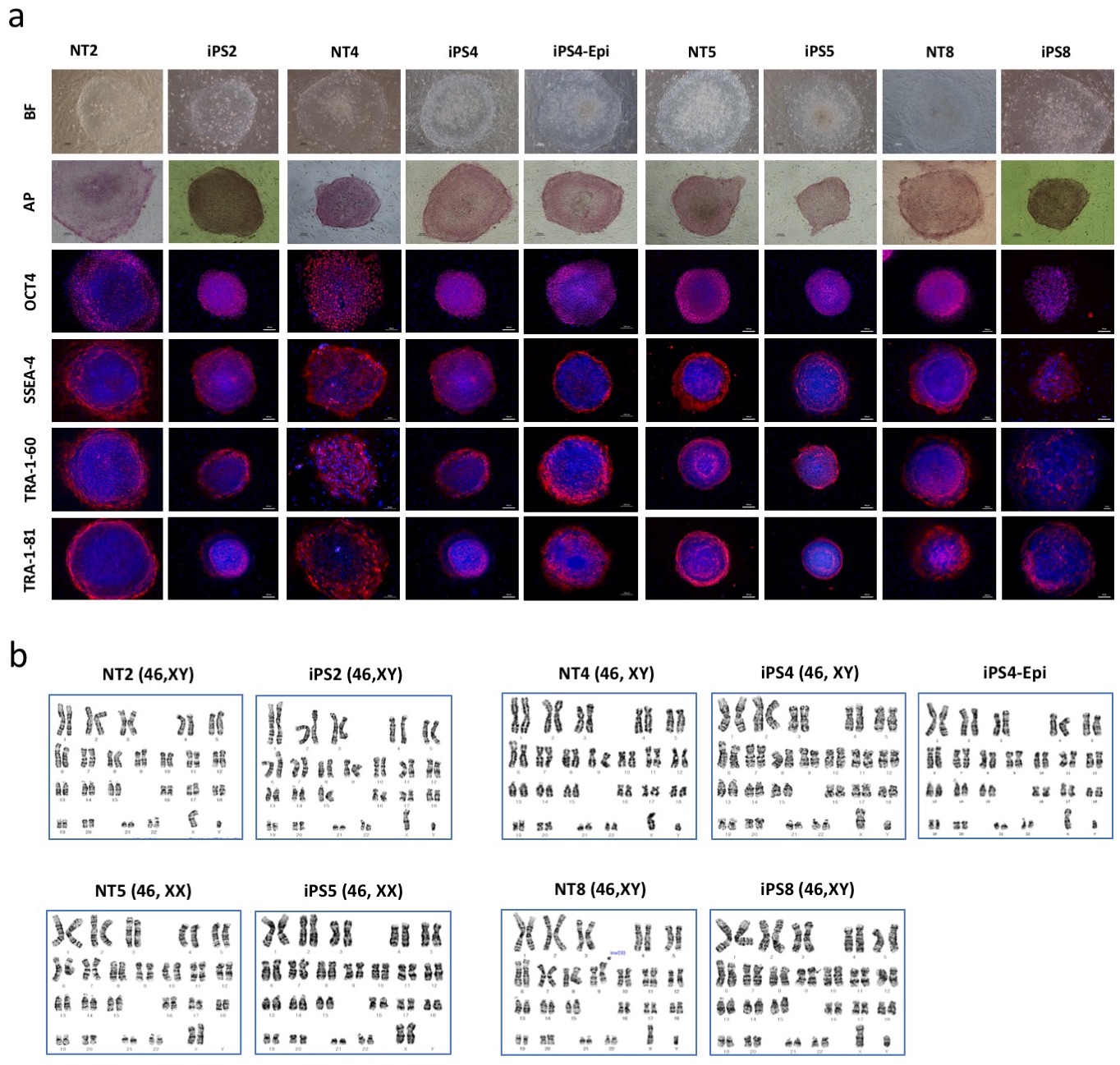
**

**Supplementary Figure 1. Generation and characterization of isogenic human NT-ESCs and iPSCs. a** Evaluation of pluripotency of human NT-ESCs and iPSCs by alkaline phosphatase staining and immunocytochemistry for pluripotency markers. Antibodies against OCT4, SSEA-4, TRA-1-60 and TRA-1-81 were used for immunocytochemistry, and endogenous alkaline phosphatase activity were assessed by AP staining kit. Scale bars are 100 µm except OCT-4, SSEA-4, TRA-1-60 and TRA-1-81 of NT2, NT4, NT8 and iPS4-Epi (50 µm). **b** Karyotyping results by G-banding.

**
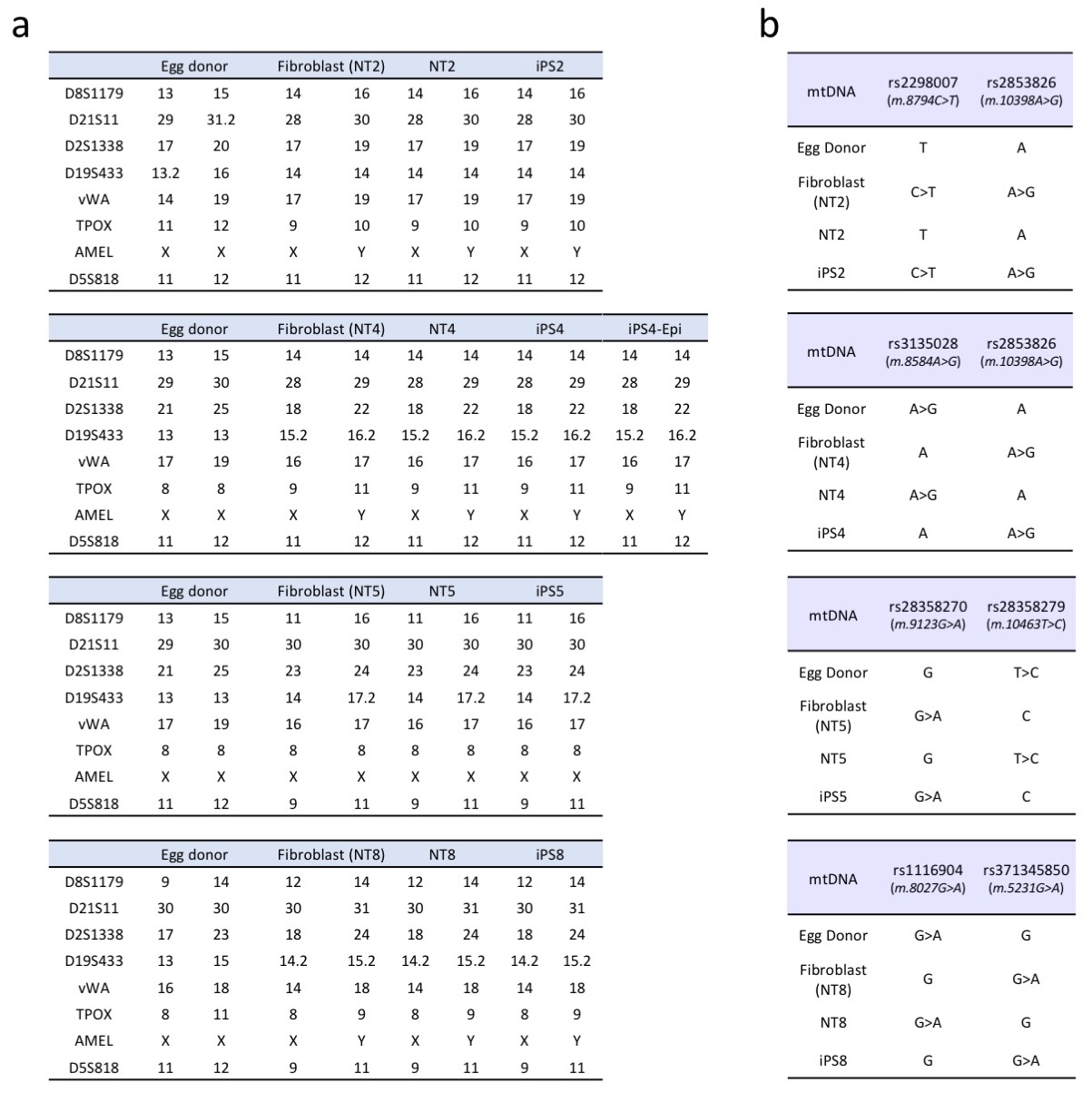
**

**Supplementary Figure 2. Genetic analysis of isogenic human NT-ESCs and iPSCs. a** Nuclear DNA genotyping was obtained using an AmpFlSTR^®^ identifier kit and an ABI 3130 genetic analyzer. There was no contribution of oocyte nuclear DNA to the NT-ESCs or iPSCs. **b** Mitochondrial DNA genotyping. The regions containing the single nucleotide polymorphism sites were amplified, sequenced, and analyzed using the SeqScape® Software. The NT-ESCs and iPSCs contained the oocyte mtDNA.

**
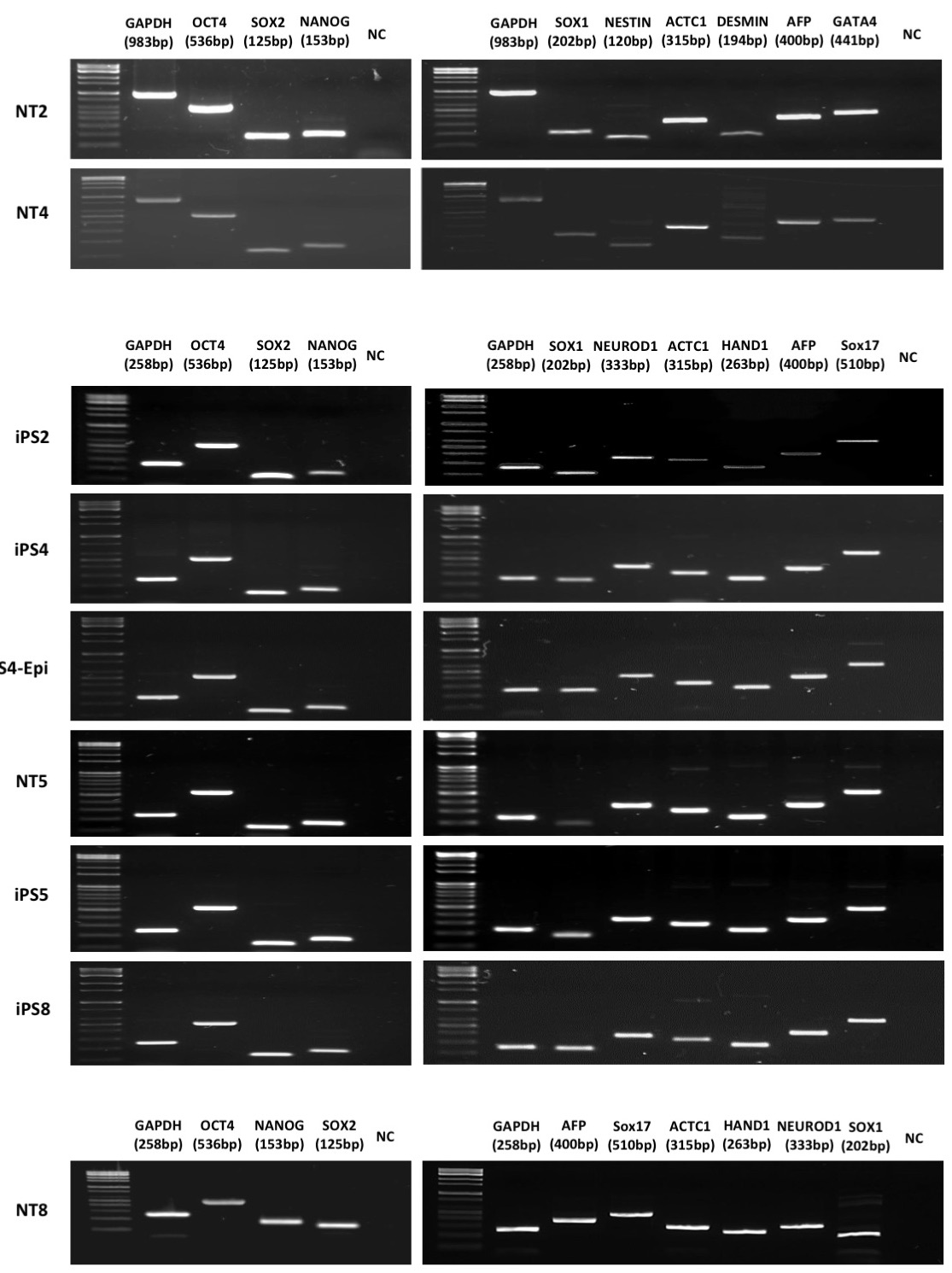
**

**Supplementary Figure 3. Pluripotency analysis of isogenic human NT-ESCs and iPSCs.** Expression of pluripotency and three-germ layer markers in NT-ESCs and iPSCs by RT-PCR. *OCT4*, *NANOG*, and *SOX2* genes were used for pluripotency markers. For three germ layer differentiation, the expression levels of *SOX1* and *NESTIN*/*NEUROD1* for ectoderm, *ACTC1* and *DESMIN*/*HAND1* for mesoderm, and AFP and *GATA4*/*SOX17* for endoderm were tested in EBs. GAPDH was used as an internal control.

**
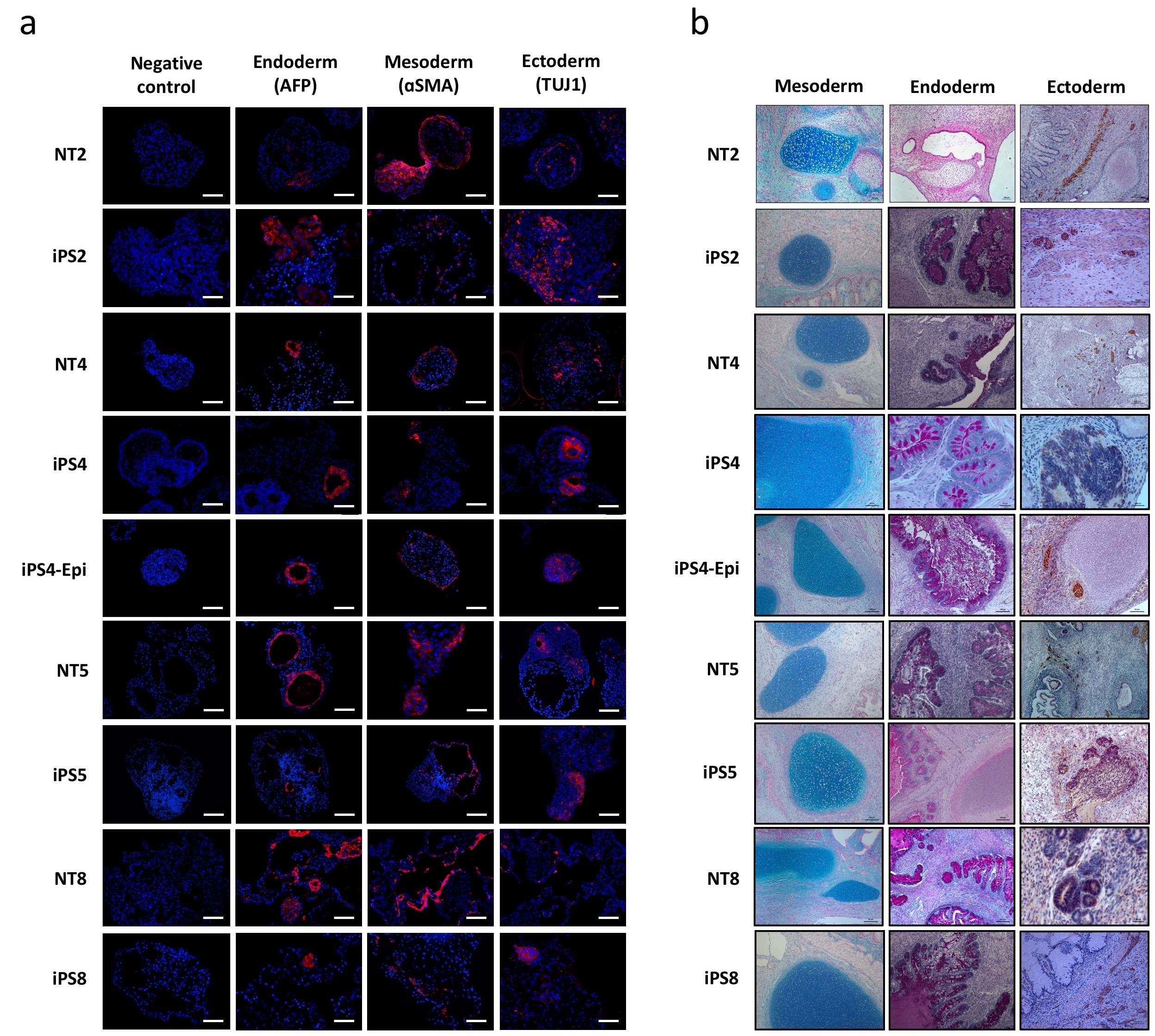
**

**Supplementary Figure 4. Teratoma formation assay of isogenic human NT-ESCs and iPSCs. a** Differentiation marker expression in EBs derived from human NT-ESCs and iPSCs by immunohistochemistry. Antibodies against AFP for endoderm, ɑSMA for mesoderm, and TUJ1 for ectoderm were used. DAPI was used for counter staining. Scale bars are 100 µm. **b** Differentiation marker expression in teratomas derived from human NT-ESCs and iPSCs. Teratoma assay confirms differentiation capacity of NT-ESCs and iPSCs into the three germ layers *in vivo*; mesoderm-derived cartilage (Alcian blue staining), endoderm-derived secretory epithelium (Periodic Acid Schiff staining), and ectoderm-derived neural epithelium with rosette (immunohistochemistry using TUJ1 antibody). Original magnification, x100.

**
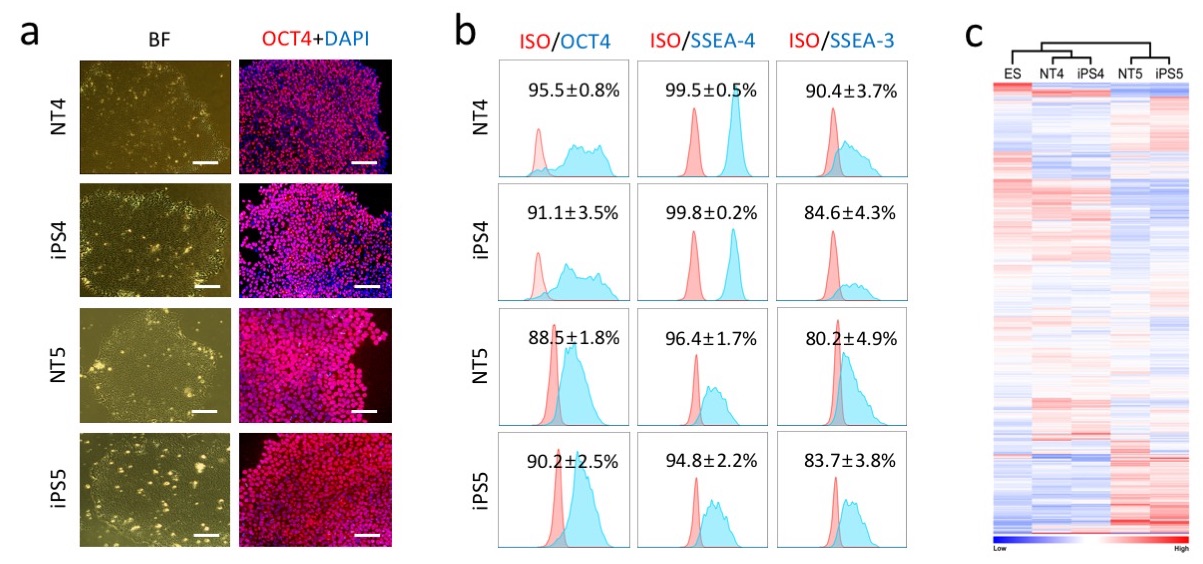
**

**Supplementary Figure 5. Similar phenotypes and transcriptional landscape between Isogenic iPSCs and NT-ESCs.** **a** Immunocytochemistry staining for OCT4 (red) in feeder-free cultures. The nuclei were counterstained with 4,6-diamidino-2-phenylindole (DAPI, blue). Scale bar, 100 *μ*m. BF, Bright field. **b** Representative histograms showing the expression of pluripotency markers (OCT4, SSEA-3/4) in feeder-free cultures. Frequencies shown in histograms indicate the mean±SD from three independent experiments. ISO, Isotype control. **c** Unsupervised hierarchical clustering of global gene expression profiles in genetically matched iPSCs and NT-ESCs. ES, human embryonic stem cells.

**
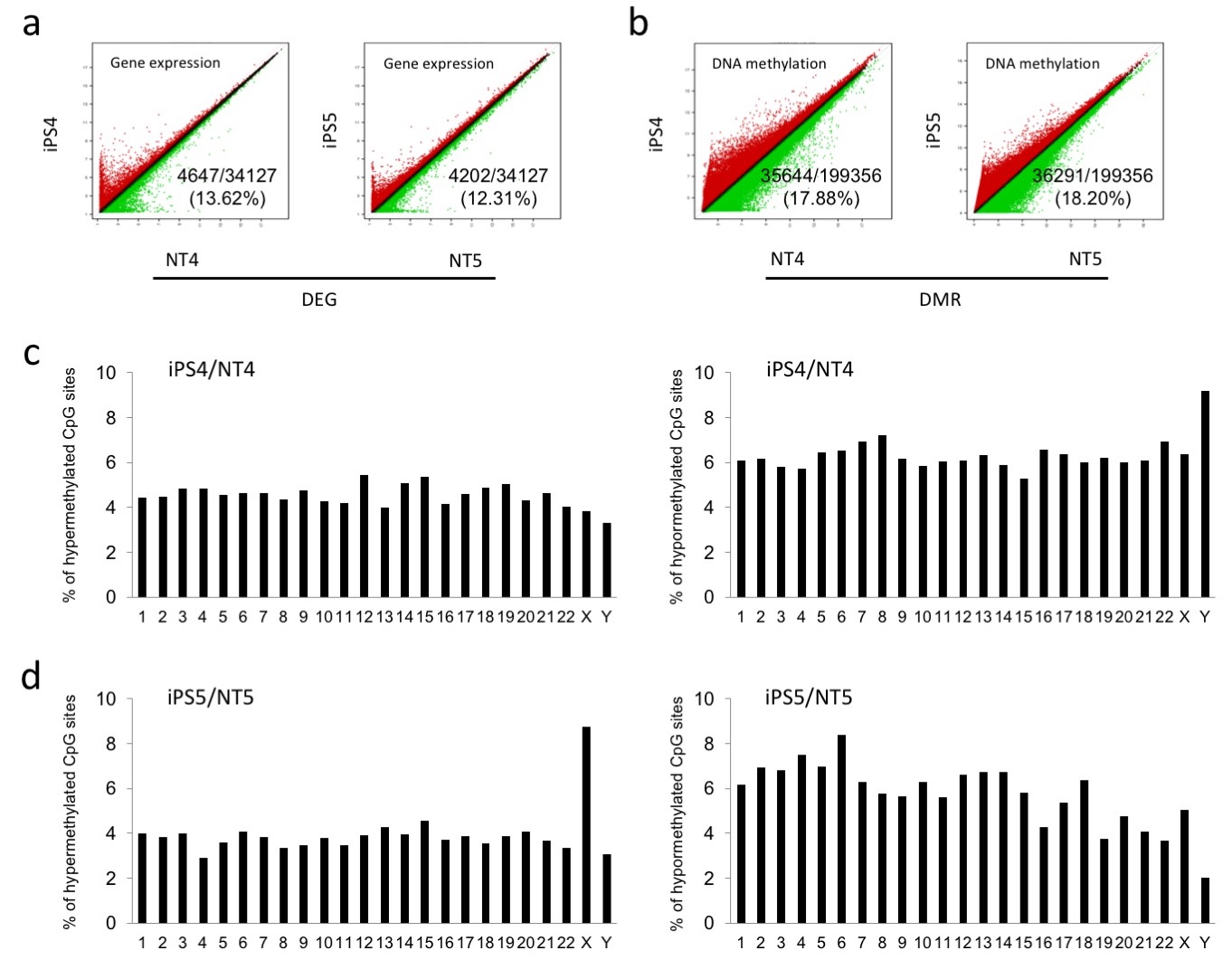
**

**Supplementary Figure 6. Transcriptomic and methylomic analyses of isogenic NT-ESCs and iPSCs. a** Scatter plots of gene expression of isogenic NT-ESCs and iPSCs in undifferentiated state by microarray analysis. Red and green dots indicate DEGs (FC>1.5, FDR<0.05). **b** DMRs between NT-ESCs and iPSCs were identified by comparing DNA methylation profiles (FC>1.5, FDR<0.05). Red and green dots indicate DMRs. **c, d** Chromosomal mapping of DMRs. Frequencies of hyper- and hypomethylated sites were shown in the graphs.

**
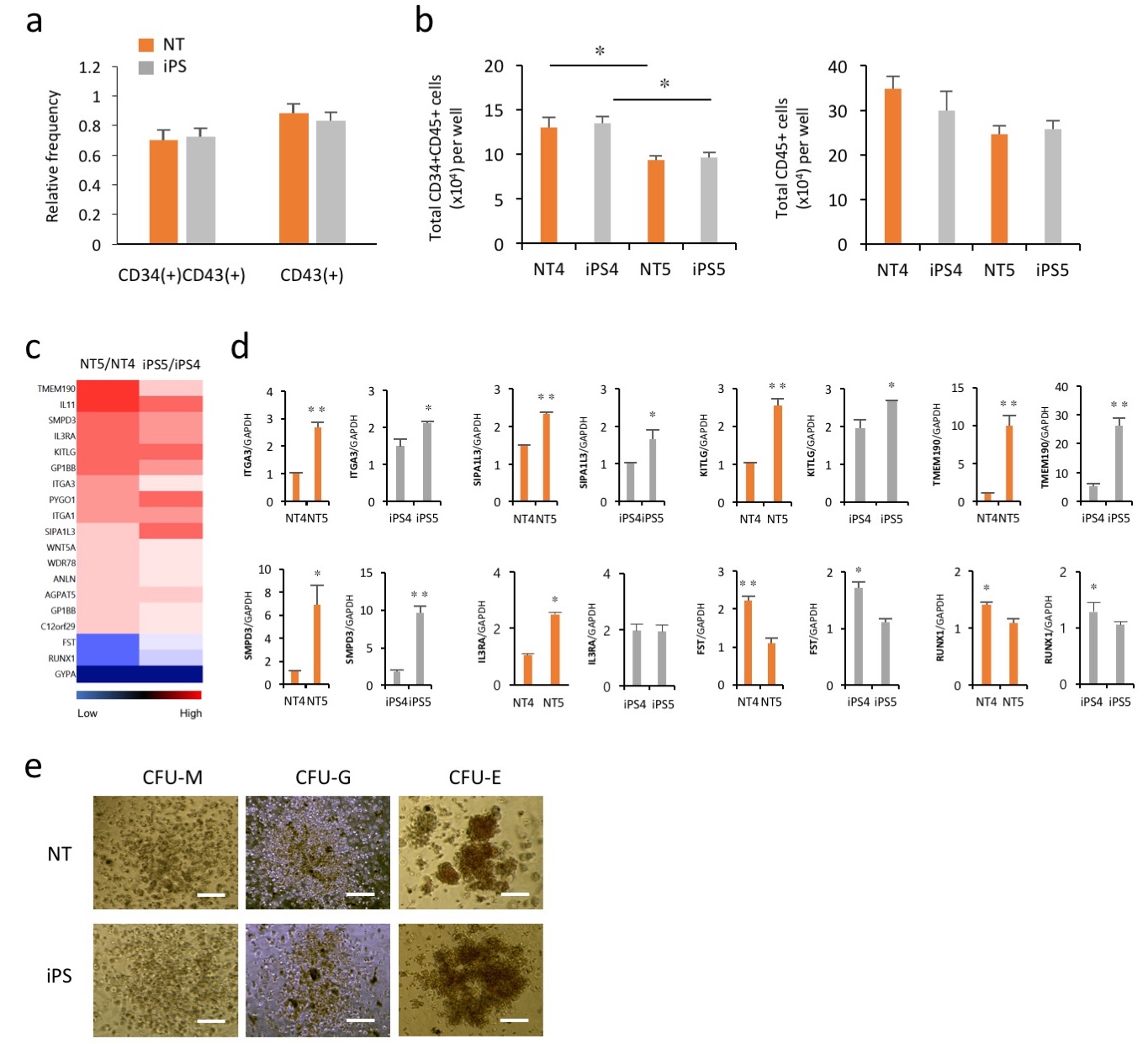
**

**Supplementary Figure 7. Hematopoietic differentiation assay of isogenic NT-ESCs and iPSCs. a** Flow cytometry analysis for committed hematopoietic progenitors (CD34+CD43+) and mature blood (CD34-CD43+) cells on day 17. **b** Total number of CD34+CD45+ and CD34-CD45+ cells on day 17. **p*<0.05. **c** A heat map showing differentially expressed hematopoietic-related genes between NT4 and NT5 or iPS4 and iPS5. **d** Verification of the transcriptomic analysis with qPCR on selected genes. **p*<0.05, ***p*<0.01. **e** Representative images of CFU subtypes derived from hematopoietic progenitors. Scale bar, 100 µm. All bars indicate the mean±SD from three independent experiments.

**
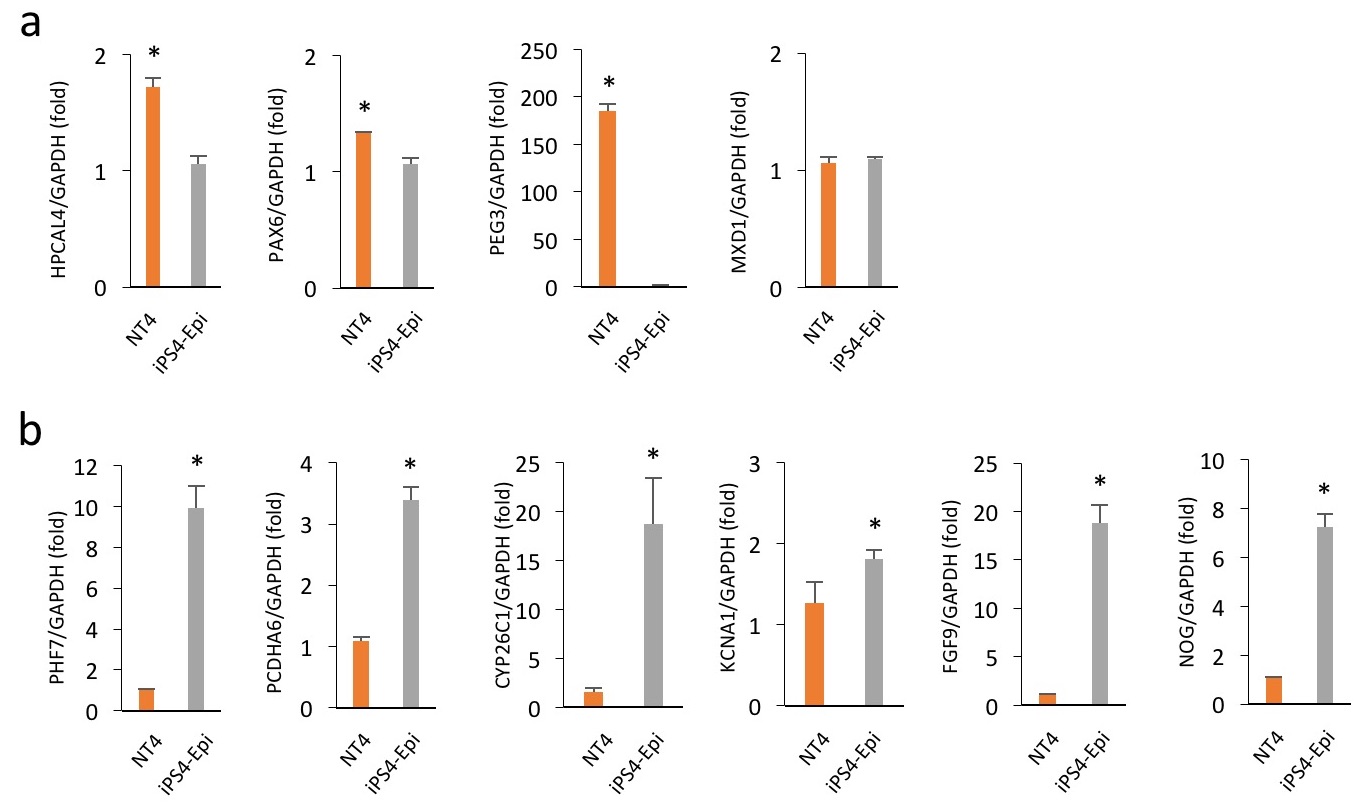
**

**Supplementary Figure 8. Comparison of hematopoietic gene expression between NT4 and iPS4-Epi.** Verification of the transcriptomic analysis with qPCR on selected up (**a**) and downregulated genes (**b**). All bars indicate the mean±SD from three independent experiments. **p*<0.05.

**Supplementary Table 1. Primer sequences for qPCR and RT-PCR**

| Gene | Sequence (5' to 3') | | |
| --- | --- | --- | --- |
| *GATA6* | F R |  | CAGCAAAAATACTTCCCCCA  ACTTGAGCTCGCTGTTCTCG |
| *NKX2.1* | F R |  | AGCACACGACTCCGTTCTC  GCCCACTTTCTTGTAGCTTTCC |
| *NEUROG2* | F R |  | CCCTGACCCCGCTGTCATCC  CCCTCCGCACCCGCAGCCAC |
| *SOX1* | F R |  | CCCGCCAGCCGCCCCGATG  CCATCTTGCGCCGCTGCCCGC |
|  | F  R |  | GTGTCCAATTGTTGGCATCTAGGTC  CAAGGAAATAAGGTGGTTGGAGCAC |
| *NESTIN* | F  R |  | TCCAGGAACGGAAAATCAAG  GCCTCCTCATCCCCTACTTC |
| *NUEROD1* | F  R |  | GTTTCTCAGGACGAGGAGCAC  TGGACAGCTTCTGCGTCTTA |
| *ACTC1* | F  R |  | CATCCTGACCCTGAAGTATCCCATC  CCCTCATAGATGGGGACATTGTGAG |
| *DESMIN* | F  R |  | TCGGCTCTAAGGGCTCCTC  CGTGGTCAGAAACTCCTGGTT |
| *HAND1* | F  R |  | AAGAGAACCAGACGCAGGAA  GGCAGGATGAACAAACACCT |
| *AFP* | F  R |  | GAAATGACTCCAGTAAACCCTGGTG  AGACTCGTTTTGTCTTCTCTTCCCC |
| *GATA4* | F  R |  | CTCTACCACAAGATGAACGGC  CATCGCACTGACTGAGAACG |
| *SOX17* | F  R |  | CGCACGGAATTTGAACAGTA  AAAACACACCCAGGACAACA |
| *OCT4* | F R |  | CTGAAGCAGAAGAGGATCAC  GACCACATCCTTCTCGAGCC |
| *GAPDH* | F R |  | TGCACCACCAACTGCTTAGC  GGCATGGACTGTGGTCATGAG |
